# Concurrent Amyand and Littre Hernias With Incidental Hodgkin Lymphoma: A Diagnostic and Surgical Challenge

**DOI:** 10.1155/cris/1130953

**Published:** 2026-06-25

**Authors:** Jia Chyi Tay, Woon Teen Sia, Siti Shakinah Sobri, Li Jie Thee

**Affiliations:** ^1^ Department of Surgery, Hospital Sultanah Bahiyah, Alor Setar, Kedah, Malaysia, moh.gov.my; ^2^ Jeffrey Cheah School of Medicine and Health Sciences, Monash University, Subang Jaya, Selangor, Malaysia, monash.edu.my; ^3^ Department of Pathology, Hospital Sultanah Bahiyah, Alor Setar, Kedah, Malaysia, moh.gov.my

## Abstract

We report a rare case of a 62‐year‐old man with a right giant inguinoscrotal hernia and a concurrent retroperitoneal mass. Preoperative imaging was inconclusive regarding the hernia sac contents and the nature of the pelvic mass. Intraoperatively, the hernia sac contained the caecum, appendix, Meckel’s diverticulum (MD) and terminal ileum—confirming the simultaneous presence of Amyand and Littre hernias. Additionally, a left‐sided retroperitoneal solid‐cystic mass was identified and excised. This case underscores the diagnostic difficulties posed by complex hernias and highlighted the surgical challenges involved in managing coexisting intra‐abdominal pathologies. Histopathological examination revealed a mesenteric cyst with infarcted tissue and an incidental diagnosis of mixed cellularity classical Hodgkin lymphoma (MCCHL) from the lymph node, adding a haematological dimension to this already rare surgical case.

**Key Clinical Knowledge**

Amyand and Littre hernias are rare and often diagnosed intraoperatively, as imaging may not reliably define hernia contents or associated retroperitoneal masses. Management of incidental Meckel’s diverticulum should be individualised, and coexisting masses may contribute to hernia progression. Histopathological evaluation remains essential to detect clinically significant incidental findings.

## 1. Introduction

Inguinal hernia is a common clinical condition, affecting 27%–43% of men and 3%–6% of women [1]. Its incidence is particularly higher in the elderly populations, due to risk factors that are associated with old age, such as constipation, chronic obstructive pulmonary disease and prostatism [2]. Amyand hernia refers to the presence of the vermiform appendix within an inguinal hernia sac, accounting for ~1% of all inguinal hernias [3]. Littre hernia involves the herniation of a Meckel’s diverticulum (MD) and is also exceedingly rare, with an unknown incidence [4]. The concurrent occurrence of both entities was first and only once reported in 2018 [5]. We present a case involving both hernia types, complicated by a large retroperitoneal mass, to highlight the difficulties in preoperative diagnosis and intraoperative management.

## 2. Case Presentation

A 62‐year‐old retired male teacher with underlying diabetes mellitus, hypertension, dyslipidaemia and chronic constipation presented with a 10‐year history of an irreducible, painless right inguinoscrotal hernia. He had no known drug or food allergies and was a non‐smoker. His functional status was good (ADL‐independent, NYHA Class I, METS > 4). His BMI was normal.

**Figure 1:**
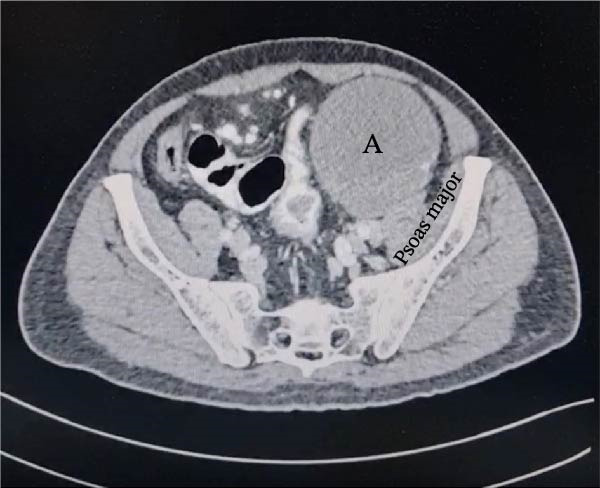
Axial CT scan showing hypodense left pelvic mass (A) measuring 9.0 cm × 7.9 cm × 9.5 cm.

**Figure 2:**
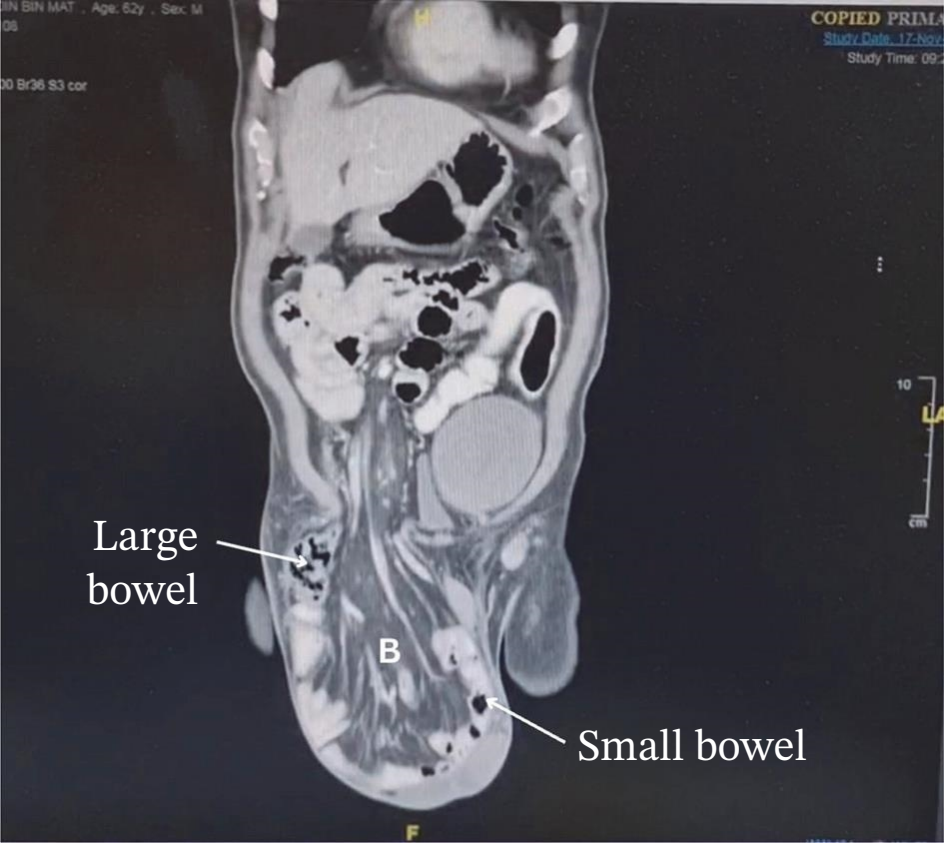
Coronal plane CT scan showing small bowel and large bowel contained in the inguinal hernia (B).

CT imaging of the abdomen and pelvis revealed a large right inguinoscrotal hernia containing bowel and mesenteric fat, without evidence of obstruction. An incidental heterogenous hypodense pelvic mass measuring 9.0 cm × 7.9 cm × 9.5 cm was noted in the left hemipelvis, compressing the urinary bladder, raising differential diagnoses of a mesenteric cyst or necrotic lymphadenopathy. (Figures 1 and 2) Colonoscopy showed no intraluminal pathology. A multidisciplinary radiology conference was organised, and laparotomy mesenteric cyst excision with right giant inguinal hernia repair was planned.

**Figure 3:**
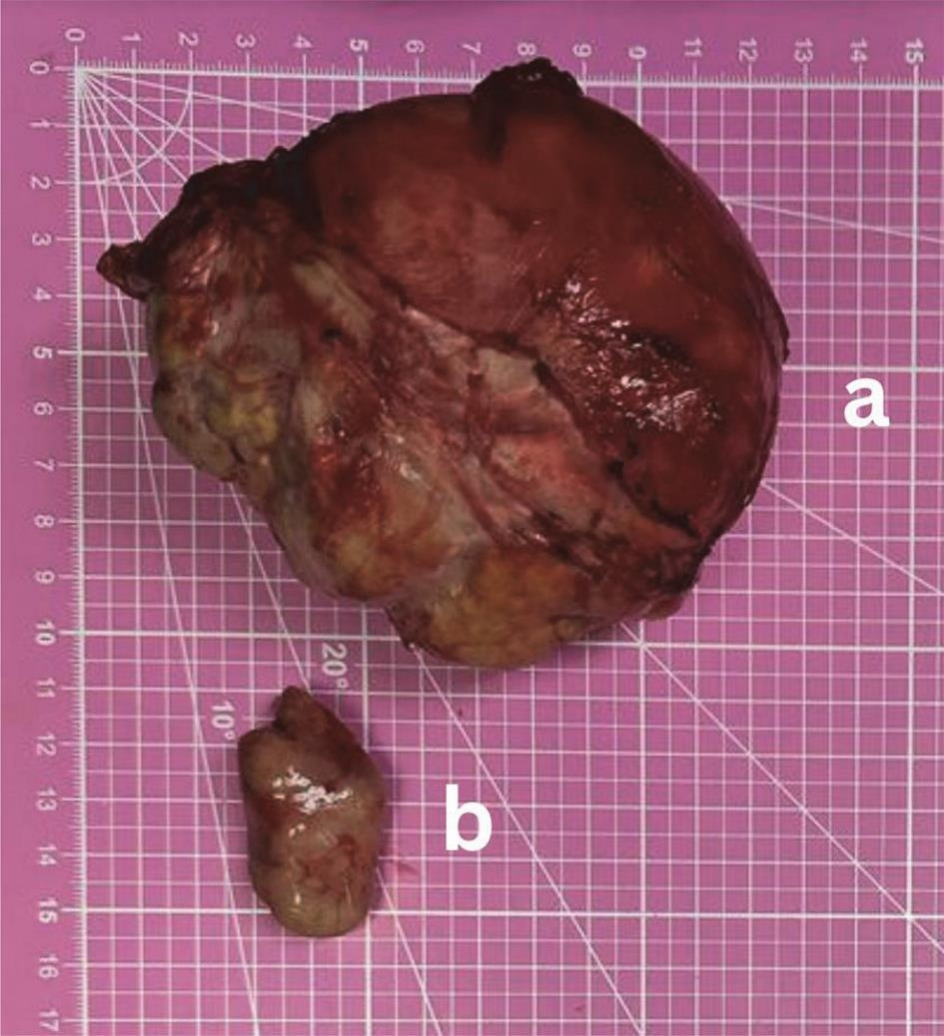
(a) Hypodense retroperitoneal mass measuring 12 cm× 11 cm; (b) lymph node posterior to the mass (2.5 cm × 4 cm) was removed.

Intraoperatively, a large solid‐cystic mass measuring 12 cm × 11 cm was identified in the left retroperitoneal space, originating near the line of Toldt. The anterior portion was cystic, while the posterior component was solid. The mass was closely related to the left ureter and external iliac vessels, but clear dissection planes were preserved. A 2.5 cm × 4 cm lymph node was found posterior to the mass and was excised. (Figure 3)

Exploration of the right inguinal region revealed an indirect inguinal hernia containing the caecum, appendix, MD and terminal ileum. Dense adhesions were present between small bowel loops, requiring adhesiolysis, during which serosal tears occurred at 50 and 60 cm from the ileocecal junction. These were repaired. The appendix was not inflamed and was preserved. The MD was broad‐based, ~2 cm in height and located 40 cm from the ileocecal junction. The external oblique aponeurosis was thinned and partially excised. The hernia was repaired and reinforced with polypropylene mesh due to a weak posterior wall.

Postoperatively, the patient was comfortable on room air, tolerated oral intake well and was able to pass flatus and urine without difficulty. There were no episodes of vomiting or abdominal pain. The patient was discharged home 1 day postoperatively.

**Figure 4:**
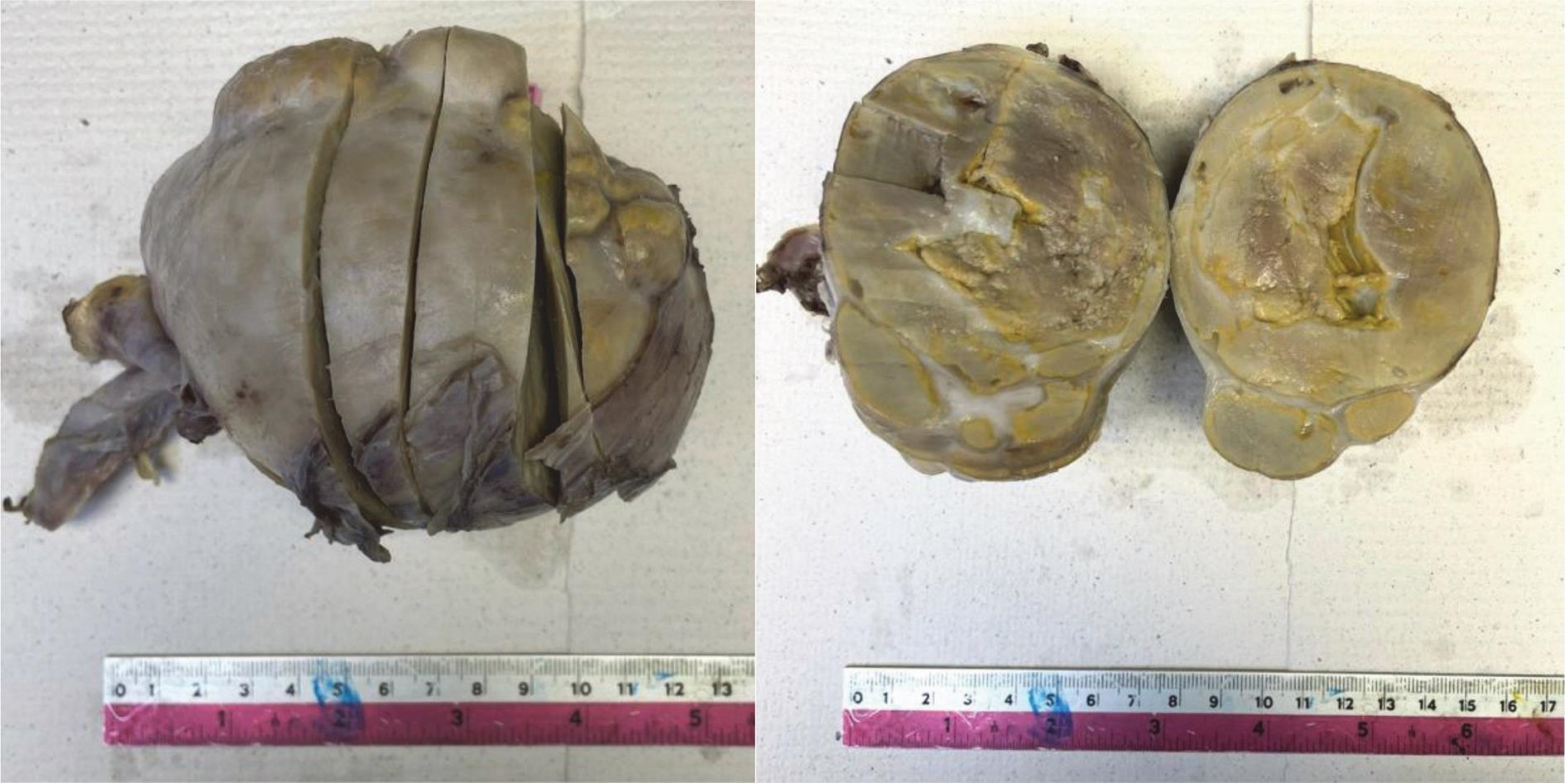
Specimen of the retroperitoneal mass: a lobulated mass with firm yellowish cut surface and extensive necrosis, measuring 110 mm × 90 mm × 50 mm.

**Figure 5:**
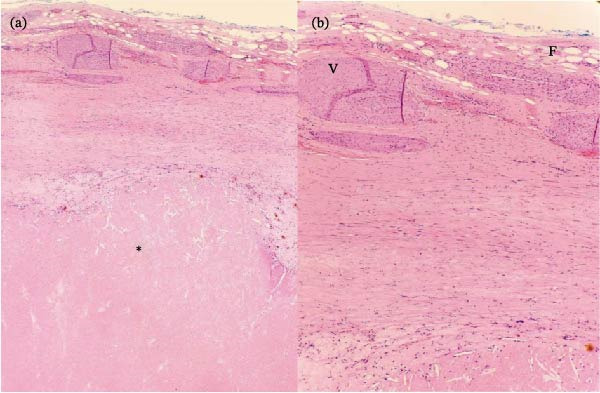
A circumscribed lesion with extensive central necrosis ( ^∗^) and minimal viable tissue at periphery containing muscular vessels (V) and fat (F). (a) H&E ×4obj and (b) H&E ×10obj.

Histopathological analysis revealed the mass to be a mesenteric cyst consisting of extensively infarcted tissue, rendering specific tissue classification inconclusive (Figures 4 and 5).

**Figure 6:**
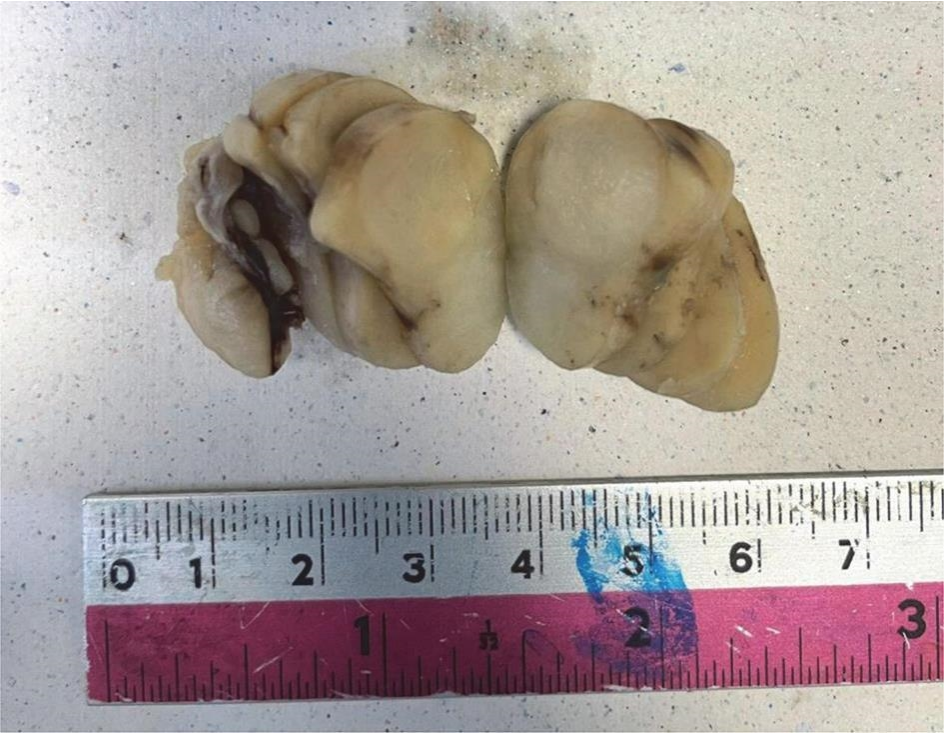
Specimen of the retroperitoneal lymph node: a lobulated mass with smooth capsule and solid greyish‐tan cut surfaces, measuring 47 mm × 22 mm× 10 mm.

**Figure 7:**
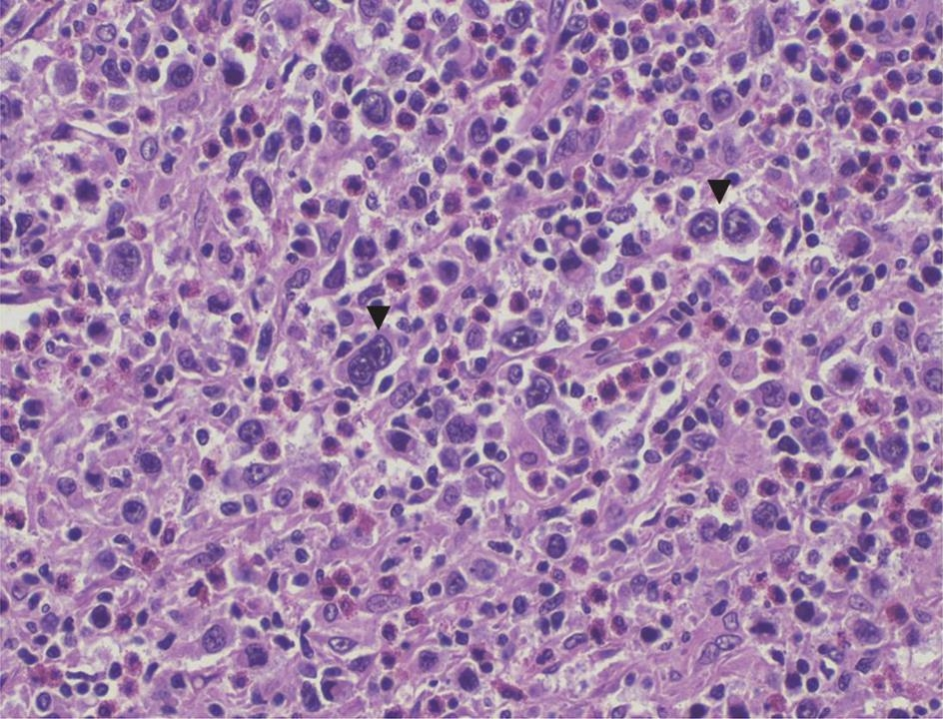
Scattered Hodgkin Reed–Sternberg (HRS, ▼) cells amidst mixed inflammatory background, composed of small lymphocytes and numerous eosinophils. (H&E ×20obj).

**Figure 8:**
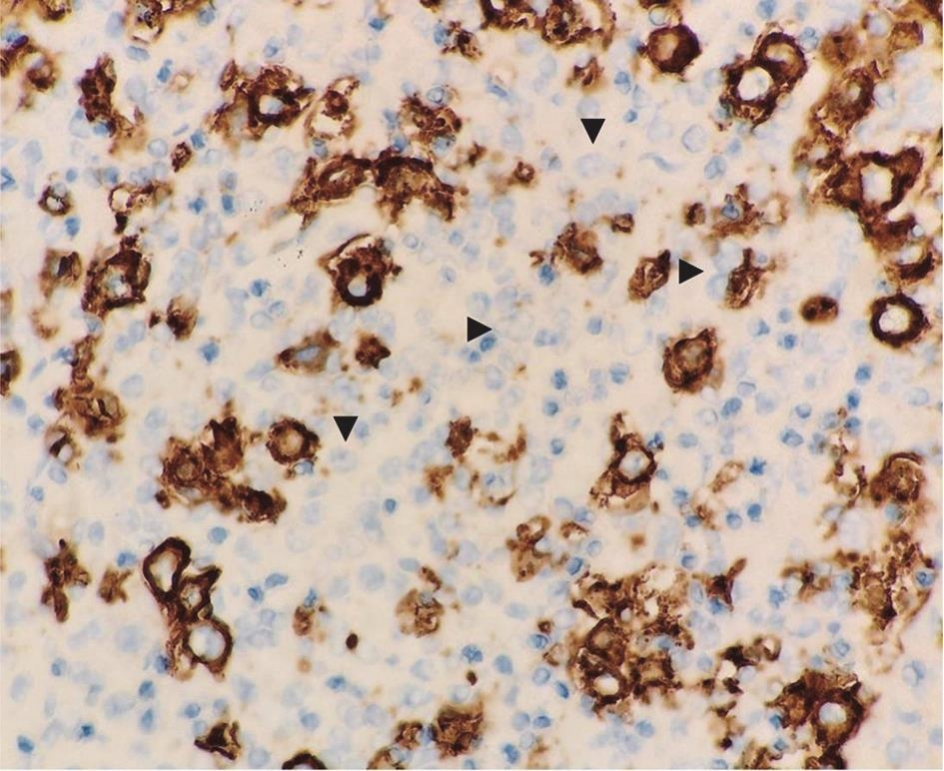
CD20 highlighted small B‐cells but negative for the large atypical cells (HRS cells, ▼)(×60 obj).

The excised lymph node demonstrated an intact capsule, containing prominent atypical Hodgkin Reed–Sternberg (HRS) positive for CD15, CD30 and PAX5 (dim) and negative for CD20, CD79a and T‐cell markers (CD2, CD3, CD5, CD7 and CD8) (Figures 6–8). The Ki‐67 index was high. This was consistent with a mixed cellularity classical Hodgkin lymphoma (MCCHL), an incidental yet clinically significant finding.

## 3. Discussion

This case represented a rare confluence of three uncommon findings: Amyand hernia, Littre hernia and a retroperitoneal mass. Each condition individually presents diagnostic challenges and requires specific surgical strategies. The concurrent occurrence of these pathologies in a single patient is extremely rare, with very few comparable cases reported in the literature.

Amyand hernia, characterised by the presence of the appendix within an inguinal hernia sac, is a known but rare differential diagnosis of irreducible inguinal hernia. It should be considered in long‐standing hernias with unusual contents [6]. The appendix in such cases might be prone to microtrauma, leading to inflammation, adhesion, incarceration, or strangulation [7, 8]. Its diagnosis is often incidental and confirmed intraoperatively, as its clinical presentations are non‐specific and dependent on the degree of peri‐appendiceal inflammation. Preoperative imaging such as ultrasound and CT scan might suggest bowel‐containing hernias but rarely identifies the appendix unless inflamed [9, 10]. In our case, the hernia aligned with Losanoff and Basson Type 1 (i.e., normal appendix in an inguinal hernia), and management was consistent with this classification [11].

Littre hernia involves a MD in the hernia sac and is even rarer [12]. Its incidence was unknown [4]. A recent systematic review identified only 53 reported cases in the literature, with the majority (73%) occurring in the groyne region. Due to its typically asymptomatic nature, it is frequently diagnosed incidentally during surgery [13]. While resection of MD is often advocated, particularly when risk factors for complications are present, management should be individualised [14, 15]. In this patient, the MD was broad‐based, non‐inflamed and without palpable abnormality. Current evidence suggests that incidental MD does not always require resection, especially in older patients, due to the low lifetime risk of complications and the potential morbidity associated with bowel resection [16–18]. Therefore, a conservative approach was adopted.

The concurrent presence of both rare hernias raised the possibility of embryological and anatomical predispositions influencing unusual hernia sac contents [7, 19]. In addition, the large retroperitoneal mass may have increased intra‐abdominal pressure and altered pelvic dynamics, potentially exacerbating the progression and irreducibility of the inguinoscrotal hernia. This underscores the importance of considering coexisting intra‐abdominal pathology in patients presenting with large or long‐standing hernias.

The retroperitoneal mass added diagnostic complexity. Mesenteric and retroperitoneal cysts, often arising from ectopic lymphatic tissue, are rare and typically asymptomatic [20]. The incidence was ~1 per 100,000 in adults [21]. Despite a high index of suspicion and appropriate imaging such as ultrasound or CT scan, a preoperative diagnosis of mesenteric cyst is only achieved in 25% of cases.[22] CT imaging in this patient suggested a pelvic mass but provided limited anatomical and tissue characterisation. Image‐guided biopsy was considered; however, it was not performed due to concerns regarding sampling error in a heterogeneous lesion, potential tumour seeding and the likelihood that definitive surgical excision would be required regardless of histological findings. Multidisciplinary input was essential. The mass’s proximity to major vessels and the ureter required careful surgical dissection. This case exemplifies the need for adaptable operative strategies, including readiness for bowel resection, stoma formation, or component separation.

Chronic constipation might have contributed to increased intra‐abdominal pressure, facilitating hernia progression and complicating surgical anatomy [23]. Additionally, the long‐standing hernia may have masked other intra‐abdominal conditions, as attention was initially focused on the hernia, with retroperitoneal pathology only addressed subsequently. Hernia repair with mesh reinforcement mirrored previously reported management strategies and aimed to prevent recurrence [5].

The incidental finding of Hodgkin lymphoma (HL) added an unexpected haematologic dimension to this case. MCCHL is the second most common subtype of classical HL and is more commonly associated with HIV infection and developing countries [24, 25]. However, our patient did not have these risk factors, making the diagnosis both unique and unexpected. Overall, this case highlights the limitations of preoperative imaging, the importance of intraoperative adaptability and the need for individualised surgical decision‐making in managing rare and complex hernia presentations.

## 4. Conclusion

This case illustrates the importance of maintaining a high index of suspicion for rare hernia contents and coexisting intra‐abdominal pathologies. The simultaneous presence of Amyand and Littre hernias with a retroperitoneal mass represented a unique diagnostic and surgical challenge. Successful outcomes relied on thorough preoperative assessment, multidisciplinary planning and a flexible, tailored intraoperative approach. This case also highlighted the value of histopathological assessment in detecting incidental but clinically important pathologies such as HL, enabling timely haematological management.

## Funding

This study did not receive any funding. Open access publishing facilitated by Monash University, as part of the Wiley ‐ Monash University agreement via the Council of Australasian University Librarians.

## Ethics Statement

This study was compliant to ethical requirement.

## Consent

Patient consented to this publication.

## Conflicts of Interest

The authors declare no conflicts of interest.

## Data Availability

Research data are not shared.
